# Nasal Carriage of Antimicrobial-Resistant Staphylococci by Fallow Deer (*Dama dama*) Taken in a Natural Park of Tuscany, Central Italy

**DOI:** 10.3390/microorganisms12112323

**Published:** 2024-11-15

**Authors:** Giulia Cagnoli, Fabrizio Bertelloni, Paolo Bongi, Silvia Piva, Marco Del Frate, Raffaele Scarpellini, Marco Apollonio, Valentina Virginia Ebani

**Affiliations:** 1 Department of Veterinary Science, University of Pisa, Viale delle Piagge 2, 56124 Pisa, Italy; giulia.cagnoli@phd.unipi.it (G.C.); valentina.virginia.ebani@unipi.it (V.V.E.); 2 Department of Veterinary Medicine, University of Sassari, 07100 Sassari, Italy; bongip73@yahoo.it (P.B.); delfratemarco@gmail.com (M.D.F.); marcoapo@uniss.it (M.A.); 3 Department of Veterinary Medical Sciences, University of Bologna, 40064 Ozzano dell’Emilia, Italy; silvia.piva@unibo.it (S.P.); raffaele.scarpellin2@unibo.it (R.S.); 4 Centre for Climate Change Impact, University of Pisa, Via del Borghetto 80, 56124 Pisa, Italy

**Keywords:** *Staphylococcus* spp., fallow deer, antimicrobial resistance, wildlife

## Abstract

Wild animals are recognized as significant reservoirs for various zoonotic pathogens, including antibiotic-resistant bacteria. This study aimed to investigate the presence of *Staphylococcus* spp. strains in fallow deer (*Dama dama*) inhabiting a natural preserve in Central Italy and to examine the phenotypic and genotypic antimicrobial resistance and the presence of some virulence genes among the isolates. During July and December 2022, nasal swabs were collected from 175 fallow deer, which were then analyzed through bacteriological cultures. In total, 176 *Staphylococcus* spp. strains were isolated and subsequently identified using MALDI-TOF mass spectrometry. *S. aureus* was the most abundant species with 66 (37.5%) strains, followed by *S. hyicus*, 34 (19.31%) strains, *S. sciuri*, 32 (18.18%) strains, *S. chromogenes*, 27 (15.34%) strains, *S. xylosus*, 11 (6.25%) strains, *S. warneri*, 5 (2.84%) strains, and *S. devriesei*, 1 (0.56%) strain. Antimicrobial susceptibility was assessed for each isolate via the agar disk diffusion method, testing a panel of 13 molecules belonging to 9 antimicrobial classes. The highest resistance rates were detected for penicillin (29.55%), rifampicin (22.73%), and amikacin (20.45%). Notably, intermediate susceptibility was observed for erythromycin (61.93%), enrofloxacin (28.41%), and ceftiofur (21.02%). Conversely, the strains exhibited particularly high susceptibility to amoxicillin/clavulanic acid (99.43%), cefoxitin (97.73%), and vancomycin (96.02%). Based on the results, 32 (18.18%) isolates were classified as multidrug-resistant (MDR). Two strains of *S. chromogenes* and one strain of *S. xylosus*, both resistant to penicillin, tested positive for the *blaZ* gene. No methicillin-resistant strains were found, and none of the isolates harbored genes associated with enterotoxin and toxic shock syndrome toxin production. This study highlights the potential role of wildlife, particularly fallow deer, as reservoirs of antibiotic-resistant *Staphylococcus* spp. strains. Such findings underscore the importance of monitoring wildlife for antimicrobial resistance, which could have implications for public health and veterinary medicine.

## 1. Introduction

*Staphylococcus* bacteria are Gram-positive cocci that form clusters resembling bunches of grapes [1]. They are widely distributed in several habitats and commonly colonize the skin and mucous membranes of animals and humans [2]. While most are harmless commensal microorganisms, some species can be pathogenic or opportunistic [3]. Staphylococci can be classified into two groups: coagulase positive (CoPS) and coagulase negative (CoNS). CoPS are highly virulent pathogenic bacteria and can cause severe diseases in animals and humans; *Staphylococcus aureus*, *Staphylococcus hyicus*, and *Staphylococcus pseudintermedius* are the main representative species in this group [4]. CoNS, on the other hand, are considered less virulent commensal bacteria, but they have become important opportunistic pathogens in recent years, being frequently involved in human and animal infections [5]. Staphylococcal infections, whether caused by CoPS or CoNS, are challenging to manage due to the widespread antimicrobial resistance acquired by these bacteria. Methicillin resistance is a major concern; it is associated with the acquisition of a gene cassette, called staphylococcal cassette chromosome (SCC), harboring the *mecA* gene or a homologous gene, conferring resistance to β-lactam antimicrobials [1,6]. Additionally, staphylococci can be resistant to other antimicrobials such as sulfamethoxazole/trimethoprim [7], erythromycin [8,9,10], gentamicin [8], tetracycline [11,12,13], and enrofloxacin [14]. However, the extent and type of resistance can vary significantly depending on the *Staphylococcus* species, hosts, and geographic location. Antimicrobial resistance is often linked to the acquisition of mobile genetic elements. Among staphylococci, several crucial genes can be transferred horizontally; notable examples include the *blaZ* genes, which confers penicillin resistance; the *ermA*, *ermB*, *ermC*, *msr(A/B),* and *mph(C)* genes, which provide resistance to macrolides; and the *aac(6′)- Ie-aph(2″)- Ia* gene, which is responsible for resistance to amikacin [15]. Finally, members of the genus *Staphylococcus* can act as important foodborne pathogens due to their capability to produce enterotoxins [16]. Over 24 types of staphylococcal enterotoxins have been identified, but the five classic enterotoxins, SEA, SEB, SEC, SED, and SEE, encoded by their respective genes (*sea*, *seb*, *sec*, *sed*, and *see*), are most frequently linked to human outbreaks. These enterotoxins are notably resistant to heat, cold, and proteolytic enzymes, making them resistant to inactivation during cooking and digestion [17].

Animals have been identified as potential asymptomatic carriers of both CoPS and CoNS, posing a risk for direct or indirect infection for humans [3,18,19,20,21]. In recent years, there has been growing interest in wildlife as a potential reservoir of antimicrobial-resistant staphylococci. A recent large-scale study conducted by Martínez-Seijas and colleagues (2023) in Spain on various animal species living in the same area, focusing on *S. aureus* only, revealed higher colonization levels in Artiodactyla and Eulipotyphla, particularly European hedgehog (*Erinaceus europaeus*) [22]. The authors found that the best sample to be examined is a nasal swab, with the exception of European hedgehogs that showed equal levels of colonization in ear, nasal, and genital samples [22]. While the European hedgehog has been identified as an important carrier of methicillin-resistant *Staphylococcus aureus* strains (MRSA) carrying the *mecC* variant [23,24], multiple studies have reported the presence of staphylococci in various wild animal species, including mammals and birds [21,25]. Investigations into animals of the Cervidae family worldwide have shown them to also be asymptomatic carriers, albeit with variations related to sample type, years, and geographic location [26,27,28,29,30]. However, much of the research on Cervidae and wildlife has focused primarily on *S. aureus* or MRSA strains.

The objective of the present study was to investigate the occurrence of *Staphylococcus* spp. in fallow deer (*Dama dama*) residing in a natural park located in an area heavily impacted by human development. Nasal swabs were collected as the initial samples. The isolated bacteria were identified at the species level, and their antimicrobial resistance profiles were analyzed. Moreover, the study examined the presence of specific resistance and virulence genes within the isolates.

## 2. Materials and Methods

### 2.1. Study Area and Sampling

Sampling was conducted in the Regional Park Migliarino—San Rossore—Massaciuccoli, located in the northwest of Tuscany, Central Italy. The park covers a surface area of approximately 23,150 hectares, encompassing dunes, forests, wetlands, and agro-forestry landscapes. The park is the home to horse and cow breeding, as well as a variety of wild mammals, including fallow deer, wild boar (*Sus scrofa*), wolf (*Canis lupus*), red fox (*Vulpes vulpes*), badger (*Meles meles*), weasel (*Mustela nivalis*), pine marten (*Martes martes*), and stone marten (*Martes foina*). Numerous wild bird species reside in the park, which also serves as a stopover habitat for migratory birds [31,32].

The population of ungulates within the Park is expanding, leading to regular implementation of culling and “capture and release in a different place” operations to manage their numbers [32].

Sampling was specifically conducted on fallow deer that were regularly culled by gamekeepers in compliance with the management plan adopted and authorized by the park authority from July to December 2022. Nasal swabs were collected from 175 deceased animals immediately after being killed but before evisceration. A single sample was taken from each animal using a sterile cotton swab inserted deeply into both nostrils. These swabs were kept refrigerated and transported to the Laboratory of Infectious Disease at the Department of Veterinary Sciences, University of Pisa, until 24 h of collection for immediate analysis. The sex and age class, based on teeth eruption and consumption [33], were recorded for each animal during sampling. Among the 175 animals sampled, 87 were females, and 88 were males. Among females, 25 were young animals under 1 year of age, four were between 1 and 2 years old, and 58 were adults over 2 years old. For males, 40 were young under 1 year, 11 were between 1 and 2 years, 25 were between 2 and 4 years, and 12 were over 4 years of age.

### 2.2. Staphylococcus spp. Isolation and Characterization

The collected swabs were placed in Brain Heart Infusion (BHI) broth (Thermo Fisher Diagnostics, Milan, Italy) with 6.5% NaCl and incubated at 37 °C for 24 h for selective enrichment. Subsequently, the broth cultures were streaked onto Mannitol Salt Agar (MSA) (Thermo Fisher Diagnostics) and Baird Parker Agar (BPA) (Thermo Fisher Diagnostics) supplemented with 2 mg/mL of oxacillin, and both were incubated at 37 °C for 24 h. Between 1 to 3 different colonies were selected from each sample and sub-cultured on Tryptic Soy Agar (TSA) (Thermo Fisher Diagnostics). The isolates were initially confirmed as *Staphylococcus* spp. with Gram staining and catalase test, then further identified at species level with matrix-assisted laser desorption/ionization time-of-flight mass spectrometry method (MALDI-TOF) using Bruker Daltonics UltrafleXtreme equipment (Bruker Daltonics, Billerica, MA, USA), and the Biotyper Real-Time Classification software v3.1 (Bruker Daltonics, Bremen, Germany). Interpretation was conducted according to Bruker standard criteria. Colonies identified with a score > 2.2 were considered at species level and were stored at −80 °C for further experiments.

### 2.3. Antimicrobial Susceptibility Tests

*Staphylococcus* spp. isolates were tested for antimicrobial susceptibility using the disk diffusion method [34]. The following antimicrobial disks (Thermo Fisher Diagnostics) were employed: penicillin (10 U), amoxicillin–clavulanate (20/10 µg), cefoxitin (30 µg), ceftiofur (30 µg), chloramphenicol (30 µg), tetracycline (30 µg), enrofloxacin (5 µg), ciprofloxacin (5 µg), gentamicin (10 µg), amikacin (30 µg), trimethoprim–sulfamethoxazole (1.25/23.75 µg), erythromycin (15 µg), and rifampicin (5 µg). Additionally, for vancomycin resistance, Minimum Inhibitory Concentration (MIC) was evaluated with the broth microdilution method [35]. Results were interpreted in accordance with CLSI and EUCAST guidelines [34,36,37]. *Escherichia coli* ATCC 25922, *Staphylococcus aureus* ATCC 25923, and *Staphylococcus aureus* ATCC 29213 were included as internal quality controls.

Staphylococcal strains that showed resistance or intermediate resistance to cefoxitin by the disk diffusion test were confirmed as methicillin-resistant by assessing the MIC to oxacillin [35]. *Staphylococcus aureus* and *S. lugdunensis* isolates showing a MIC ≥ 4 μg/mL were considered resistant, whereas *S. pseudintermedius*, *S. schleiferi,* and CoNS isolates showing a MIC ≥ 0.5 μg/mL were classified as resistant [38].

Based on their antimicrobial resistance profiles, staphylococcal strains were categorized as multidrug-resistant (MDR), extensively drug-resistant (XDR), and pandrug-resistant (PDR), following guidelines proposed by Magiorakos and colleagues [39].

### 2.4. Detection of Virulence and Antimicrobial Resistance Genes

DNA was extracted from overnight cultures using the Quick-DNA Miniprep Plus Kit (Zymo Research, Irvine, CA, USA), according to the manufacturer’s instructions.

*Staphylococcus* spp. isolates were then tested for the presence of enterotoxins encoding genes (*sea*, *seb*, *sec*, *sed*, *see*) as well as the Toxic Shock Syndrome Toxin 1 (*tst1*) encoding gene [40].

Isolates that were resistant to oxacillin in phenotypic tests were further evaluated for the presence of the genes *mecA*, *mecC,* and *blaZ*. The *blaZ* gene was also investigated in staphylococci resistant or intermediate to penicillin. Isolates that were not susceptible to erythromycin were tested for the presence of genes *ermA*, *ermB*, *ermC*, *msr(A/B),* and *mph(C)* [41,42]. Additionally, isolates resistant to amikacin were tested for the presence of the gene *aac(6′)- Ie-aph(2″)- Ia* [43].

Primers and protocols from the literature were used and are detailed in Table 1. PCR assays were performed in an automated thermal cycler (SimpliAmp™Thermal Cycler, Applied Biosystems, Waltham, MA, USA), using DreamTaq Hot Start Green Master Mix (Life Technologies Italia, Milan, Italy). Sterile ultrapure water was used as negative control, while DNA from previously characterized field strains served as a positive control. The PCR products were run on a 1.5% agarose gel stained with ethidium bromide at 100 V for 45 min. The gels were then observed under UV light, with a 100 bp DNA Ladder Ready to Load (Solis BioDyne, Tartu, Estonia) used as DNA marker.

### 2.5. Statistical Analyses

The obtained results were analyzed using the Chi-square (X^2^) test. This statistical test was utilized to examine the distribution of *Staphylococcus* species in relation to sex and age of deer. Additionally, it was used to compare the antimicrobial resistance of staphylococci based on bacterial species, as well as sex and age of deer. The statistical significance threshold was set at a *p*-value ≤ 0.05.

## 3. Results

### 3.1. Isolation and Typing of Staphylococci

Staphylococci were isolated and correctly identified from 169 out of 175 samples, resulting in a success rate of 96.57%. One single isolate was obtained in pure culture from all positive samples, but it was possible to obtain two different isolates from 7/169 samples. In total, 176 *Staphylococcus* spp. isolates were isolated and further analyzed, all obtained from MSA as the selective medium for methicillin-resistant staphylococci did not yield any results.

The isolated staphylococci were identified as belonging to 7 species (Table 2): *Staphylococcus aureus* (66/176 isolates—37.50%), *Staphylococcus hyicus* (34/176 isolates—19.32%), *Staphylococcus sciuri* (32/176 isolates—18.18%), *Staphylococcus chromogenes* (27/176 isolates—15.34%), *Staphylococcus xylosus* (11/176 isolates—6.25%), *Staphylococcus warneri* (5/176 isolates—2.84%), and *Staphylococcus devriesei* (1/176 isolates—0.57%). There were no significant differences in *Staphylococcus* species between males and females (*p* > 0.05), but some differences were observed in relation to age (Table 2). Specifically, *S. hyicus* was more commonly found in samples from young animals (*p* = 0.01719735), *S. sciuri* in samples from juvenile animals (*p* = 0.00013006), and *S. chromogenes* in samples from adult animals (*p* = 0.01523744).

### 3.2. Antimicrobial Resistance 

Regarding antimicrobial resistance, most antimicrobials were active against more than 80% of the isolates, as shown in Table 3. The most effective antimicrobials were amoxicillin–clavulanate and cefoxitin, with 99.43% and 97.73% of susceptible isolates, respectively. However, a low susceptibility was observed for erythromycin, with only 32.95% of isolates being susceptible. In this case, the majority of the strains (61.93%) showed intermediate susceptibility. A reduced susceptibility was also noted for fluoroquinolones, with 72.16% of strains susceptible to ciprofloxacin and 60.80% of strains susceptible to enrofloxacin. Once again, most of the strains fell into the intermediate category. High levels of resistance were observed for penicillin, with 29.55% of isolates being resistant; rifampicin, with 22.73% of resistant isolates, and amikacin, with 20.45% of resistant isolates.

Some differences in antimicrobial resistance were observed among different *Staphylococcus* species. Penicillin resistance was more frequent (*p* = 0.00001282) in *S. sciuri* (59.38% of resistant isolates), *S. xylosus* (54.55% of resistant isolates), and *S. hyicus* (41.18% of resistant isolates). Ceftiofur resistance was only detected in *S. sciuri* (15.63% of resistant isolates) and *S. hyicus* (8.82% of resistant isolates) (*p* = 0.01530269). Rifampicin resistance was more common (*p* = 0.00000744) in *S. sciuri* (56.25% of resistant isolates), *S. aureus* (25.76% of resistant isolates), and *S. xylosus* (18.18% of resistant isolates).

The high percentage of intermediate results for erythromycin was mainly due to *S. sciuri* (75.00% of intermediate isolates), *S. xylosus* (72.73% of resistant isolates), and *S. aureus* (71.21% of resistant isolates) (*p* = 0.00002716). Low susceptibility to enrofloxacin was detected mainly in *S. sciuri* (25.00% of susceptible isolates), *S. warneri* (40.00% of susceptible isolates), and *S. aureus* (54.55% of susceptible isolates) (*p* = 0.00000691). The same species were also less susceptible to ciprofloxacin: *S. sciuri* (53.12% of susceptible isolates), *S. warneri* (60.00% of susceptible isolates), and *S. aureus* (62.12% of susceptible isolates) (*p* = 0.00075759).

No statistical differences were found in relation to bacterial species for resistance to the other antimicrobial (*p* > 0.05). No statistically significant differences were found comparing the isolates in relation to animal sex (*p* > 0.05). However, higher percentages of strains showing intermediate susceptibility to ceftiofur (*p* = 0.00047987) and chloramphenicol (*p* = 0.01187286) were observed in juvenile deer (60.00% and 33.33% of intermediate strains, respectively), than in adult (15.96% and 12.77% of intermediate strains, respectively), and young animals (19.40% and 5.97% of intermediate strains, respectively). In the case of erythromycin, a higher proportion of strains with intermediate and resistant to this antimicrobial were identified in juvenile deer (73.33% and 13.33% strains, respectively), compared to adult (65.96% and 6.38% of strains, respectively), and young deer (53.73% and 1.49% of strains, respectively) (*p* = 0.01791709). Additionally, a higher percentage of staphylococci strains resistant to gentamicin (*p* = 0.00706568), amikacin (*p* = 0.00061925), and rifampicin (*p* = 0.00000571) were found in juvenile deer (13.33%, 46.63% and 66.67% of strains, respectively), compared to adult deer (8.51%, 25.53%, and 25.53% of strains, respectively), and young deer (2.99%, 7.46%, and 8.96% of strains, respectively). No statistical differences were observed among the other antimicrobials in relation to the age of the animals (*p* > 0.05).

None of the isolates tested showed resistance to vancomycin. Seven isolates (7/176—3.98%) showed intermediate susceptibility: five *S. aureus* strains, one *S. chromogenes* strain, and one *S. hyicus* strain.

Out of the four isolates resistant to cefoxitin, none were resistant to oxacillin through the MIC test.

Overall, 37/176 (21.02%) of the isolates were susceptible to all antimicrobials tested, and 85/176 (48.30%) were resistant to one or more antimicrobials. Moreover, 54/176 (30.68%) were resistant to any antimicrobials but intermediate to one or more antimicrobials. The resistant strains were found to be resistant from one to nine antimicrobials across one to seven different classes (Table 4). Among these, 32/176 (18.18%) were classified as MDR, while XDR or PDR strains were not identified. *Staphylococcus sciuri* was the most resistant species, with 14/32 (43.75%) MDR isolates (*p* = 0.00309805). No statistical differences were found among the other species tested. There were no statistical variances in the occurrence of MDR strains based on the sex of the animals (*p* > 0.05). However, MDR strains were more prevalent in juvenile (46.27%) and adult (22.34%) deer compared to young animals (5.97%) (*p* = 0.00033698).

### 3.3. Molecular Analyses

None of the strains resistant to cefoxitin was positive for *mec* genes. Out of the 52 staphylococci phenotypically resistant to penicillin, 3 (5.55%) tested positive for the *blaZ* gene (Table 4). Furthermore, none of the strains resistant and intermediate to erythromycin were positive for *ermA*, *ermB*, *ermC,* and *msr(A/B)* genes. However, two strains, both with intermediate susceptibility, were positive for the *mph(C)* gene. Finally, none of the isolates resistant to amikacin were positive for the gene *aac(6′)- Ie-aph(2″)- Ia*.

None of the investigated virulence genes were identified in any of the tested strains.

## 4. Discussion

Wildlife may serve as a reservoir and source of various pathogenic bacteria, including staphylococci. In this manuscript, we investigated the infection level in fallow deer and identified and characterized the *Staphylococcus* species colonizing these animals.

A nasal swab was selected for sample collection based on findings from several studies indicating that this method is particularly effective for isolating *Staphylococcus* spp., especially *Staphylococcus aureus* [22,25,30].

Our data confirm that deer serve as significant carriers of staphylococci, with a striking positivity rate of 96.57%. While the findings of this investigation can only be partially compared to existing studies, primarily because most have concentrated on *S. aureus* exclusively, it is evident that these bacteria are widely disseminated within deer populations. In Spain, the prevalence of nasal carriage of *S. aureus* in red deer (*Cervus elaphus*) ranges between approximately 20 and 40% [22,25]. Meanwhile, in Eastern European countries such as Slovakia, Hungary, and Poland, *Staphylococcus* species were detected in 1.53% of fecal samples from red deer [27]. An investigation conducted in Austria, Germany, and Sweden revealed that *S. aureus* was recovered from 8.69% of roe deer (*Capreolus capreolus*), 50.00% of sika deer (*Cervus nippon*), 37.50% of red deer (*Cervus elaphus*) and 30.00% of fallow deer (*D. dama*), utilizing various sample types [21]. In Portugal, nasal swabs taken from fallow deer and red deer yielded positive results for *S. aureus* in 21.7% and 53.7% of cases, respectively [28]. Lastly, a recent study in Italy reported an *S. aureus* positivity rate of 81.3% among red deer, which increased to 90% when considering only nasal swabs [30].

In the analysis of staphylococcal species, *S. aureus* emerged as the most prevalent, accounting for 37.50% of the isolates, a prevalence consistent with findings from previous studies [21,28,30]. Notably, our results indicate the presence of various other species, including both CoPS and CoNS, thereby underscoring and confirming the role of fallow deer as nasal carriers of staphylococci. Limited research has addressed the diversity of the staphylococci population found in the nasal cavity of deer. Notably, two studies conducted in Romania identified several species in the nasal cavity of fallow deer, including *S. aureus*, *Staphylococcus lentus*, *S. sciuri*, *Staphylococcus vitulinus*, *S. xylosus,* and *Staphylococcus hominis* [29,46]. Additionally, *S. vitulinus* was also detected in nasal swabs from red deer in Spain, although the focus of that study was specifically on methicillin-resistant strains [47].

The results of the present study highlighted notable differences in species distribution among various age groups of animals. Specifically, *S. hyicus* was predominantly isolated from young animals, *S. sciuri* from juvenile animals, and *S. chromogenes* from adult animals. A comprehensive comparison with existing studies on wild animals and staphylococcal species other than *S. aureus* is challenging due to the limited number of relevant publications. For instance, Ramos et al. (2022) did not identify any differences in the distribution of *S. aureus* between juvenile and adult red deer [28], a finding that aligns with similar research conducted in Northern Italy on the same deer species [30]. These results correspond with our findings, which demonstrate an even distribution of *S. aureus* across animals of all age groups. Contrastingly, some studies have reported varying rates of nasal colonization by *S. aureus* in relation to age, observed both in domestic animals such as pigs and rabbits [48,49], as well as in humans [50]. The patterns observed in our study may be linked to physiological differences associated with the ages of the host. However, hypothesizing a potential correlation between age-related animal behavior and the distribution of staphylococcal species poses significant challenges. This topic requires more extensive investigation for further clarification. The varying prevalence of different staphylococcal species among age groups provides insights into the differing levels of antimicrobial resistance observed in age-related strain categories, as will be discussed further below.

The second parameter assessed was the antimicrobial resistance of the isolated strains. Our findings indicate that the majority of the antimicrobials tested demonstrated in vitro activity against over 80% of the isolates.

β-lactams are antimicrobials commonly employed in human medicine as first- and second-line treatments [51], and they are frequently used in veterinary medicine too. In our study, amoxicillin–clavulanate and cefoxitin showed the best results, with antimicrobial susceptibility rates of 99.43% and 97.73% among isolates, respectively. Comparisons with other studies prove challenging due to the predominant focus on methicillin-resistant staphylococci in the existing literature; nevertheless, our findings are consistent with several related surveys. Notably, Porrero et al. (2014) did not report any cefoxitin-resistant *S. aureus* strains in red deer across Spain [25], while Ramos et al. (2022) identified a low percentage of cefoxitin-resistant *S. aureus* strains in fallow deer and red deer in Portugal [28]. In our study, a relatively high percentage of strains (25.57%) exhibited resistance or intermediate susceptibility to ceftiofur. To our knowledge, no other studies have reported on the susceptibility of staphylococci isolated from deer species to this antimicrobial. This gap may be attributed to the fact that ceftiofur is not utilized in human therapy; however, it is a broad-spectrum, third-generation cephalosporin widely used in veterinary medicine for treating Gram-positive and Gram-negative infections in food-producing animals and, especially, horses [52,53]. As a result, it is infrequently tested in vitro against potentially zoonotic pathogens. Monitoring ceftiofur resistance would be prudent, given evidence that the use of this antimicrobial can contribute to resistance against ceftriaxone, a molecule used in human medicine [54]. Penicillin emerged as the antimicrobial with the highest percentage of resistant strains in our study, accounting for 29.55% of isolates. As stated, β-lactams, particularly penicillin, are frequently utilized as first-line antimicrobials in both humans and veterinary medicine, leading to a prevalent occurrence of resistance to these drugs. Our findings align with those of other studies in this field. Martínez-Seijas et al. (2023) reported a 32.8% prevalence of penicillin-resistant *S. aureus* in samples collected from various wild animals in Spain [22]. Similarly, Ramos et al. (2022) found that 27.1% of *S. aureus* isolated from wild ungulates in Portugal exhibited penicillin resistance, making it the most common antimicrobial associated with phenotypic resistance [28]. Porrero et al. (2014) also identified a 19.29% rate of penicillin-resistant *S. aureus* in samples collected from Red deer in Spain [25]. In contrast, Lauková et al. (2020) did not detect ampicillin-resistant CoPS and CoNS in red deer samples from Poland [55]. The resistance to penicillin in staphylococci is often linked to the presence of the *blaZ* gene, as confirmed in strains isolated from wildlife, too [22]. Additionally, it can be associated with the presence of *mec* genes conferring methicillin resistance [15]. In our investigation, only 5.55% of penicillin-resistant strains carried the *blaZ* gene, and no *mec*-positive strains were identified. This finding is unexpected; however, the literature suggests that β-lactam resistance mechanisms beyond the presence of *blaZ* and *mec* genes can occur, even though they are relatively rare [15]. Furthermore, in our study, penicillin resistance was notably more prevalent in certain species, including *S. sciuri*, *S. xylosus*, and *S. hyicus*. Strains from these species accounted for 39 out of 52 (75.00%) of the resistant isolates. Given that most research has predominantly concentrated on *S. aureus*, it is plausible that alternative resistance mechanisms may have evolved within these species, as suggested by other researchers studying CoNS isolated from wildlife [56].

A significant percentage of strains exhibited resistance to rifampicin, specifically 22.73%. This resistance among staphylococci is primarily attributed to mutations in the *rpoB* gene, which encodes for RNA polymerase [15]. There is a limited body of research evaluating rifampicin resistance in staphylococci isolated from deer. For instance, a study conducted in Spain found no rifampicin-resistant staphylococci among 57 strains of *S. aureus* isolated from red deer [25]. Conversely, another survey in Spain reported the presence of three rifampicin-resistant strains among ten *mecA*-positive staphylococci [47].

Our findings showed that a considerable percentage (20.45%) of strains exhibited resistance to amikacin, an antimicrobial in the aminoglycosides class. The primary mechanism of aminoglycoside resistance in staphylococci is the production of transferase enzymes. Notably, the acetyltransferase/phosphotransferase enzyme AAC(6′)-Ie/APH(2″)-Ia, characterized by its two-domain structure, mediates resistance not only to amikacin but also to gentamicin, tobramycin, kanamycin, and netilmicin. The role of other transferases in conferring amikacin resistance remains uncertain [15]. Importantly, all our strains tested negative for the *aac(6′)- Ie-aph(2″)- Ia* gene, which aligns with the low percentage (6.82%) of gentamicin-resistant strains observed in our study. While there are alternative mechanisms of aminoglycoside resistance, these are less common and not typically transferable. In a study conducted in Romania, researchers did not detect amikacin-resistant staphylococci among strains isolated from fallow deer, identifying only one gentamicin-resistant *Staphylococcus* spp. out of 37 samples [29]. Similarly, Lauková and colleagues found no aminoglycoside-resistant CoNS in roe deer in Poland, testing tested gentamicin, neomycin, and tobramycin [55]. In Spain, two independent studies conducted between 2009 and 2011 and between 2021 and 2022 found no aminoglycoside-resistant *S. aureus* in red deer [22,25]. Furthermore, Martínez-Seijas and collaborators did not detect the *aac(6′)- Ie-aph(2″)- Ia* gene in the stains they tested [22]. In contrast, Rey Pérez and colleagues reported the isolation of two *mecA*-positive *S. vitulinus* strains from red deer in Spain, both of which were resistant to gentamicin [47].

The majority of tested isolates displayed intermediate susceptibility for fluoroquinolones (enrofloxacin and ciprofloxacin) and erythromycin. Specifically, enrofloxacin is an antimicrobial agent exclusively used in veterinary medicine [57], while ciprofloxacin is also utilized in human therapy and is categorized among Critically Important Antimicrobials for Human Medicine [51,54]. In *Staphylococcus* spp., resistance to fluoroquinolones is primarily attributed to genetic mutations in the genes encoding gyrase and topoisomerase. Although the overproduction of efflux pumps has also been reported, it is less frequently encountered [15]. In this study, a limited number of strains exhibited full resistance; however, a relatively high percentage of strains demonstrated intermediate susceptibility: 19.89% for ciprofloxacin and 28.41% for enrofloxacin. These results contrast with findings from other investigations conducted on deer. For instance, Tirziu and colleagues reported a higher percentage (81.08%) of susceptible staphylococci to enrofloxacin in fallow deer in Romania [29]. Additionally, no fluoroquinolones-resistant strains were detected in red deer in Spain across two separate studies [22,25]. Moreover, over 60% of the isolates tested in this study showed intermediate susceptibility to erythromycin. Various mechanisms of resistance to macrolides exist in *Staphylococcus*, with the most prevalent being the modification of the target site for the antimicrobial, mediated by several mobile genes. We evaluated the intermediate and resistant strains for the most common resistance genes (*ermA*, *ermB*, *ermC*, *msr(A/B)*, and *mph(C)*), but only two intermediate strains tested positive for the *mph(C)* gene. Our findings diverge from those of other surveys conducted on deer. In a similar study in Romania, authors identified a percentage of erythromycin-resistant strains of 16.21% and a percentage of intermediate strains of 8.11%. They also tested macrolides other than erythromycin, revealing a high percentage of staphylococci resistant to at least one antimicrobial in this class [29]. Conversely, all the staphylococci isolated during a survey in Poland focused on roe deer were found to be susceptible to erythromycin [55]. Similarly, three distinct studies on red deer in Spain reported no erythromycin-resistant staphylococci [22,25,47]. 

In terms of chloramphenicol, tetracycline, and trimethoprim–sulfamethoxazole, the majority of the tested isolates exhibited susceptibility, with only a small percentage demonstrating resistance. Notably, no vancomycin-resistant strains were identified, and merely 3.98% of isolates displayed intermediate resistance. Furthermore, no methicillin-resistant staphylococci carrying *mec* genes were detected. These findings are consistent with previous studies conducted on deer in Europe [22,25,29,47].

Approximately 50% of the tested staphylococci exhibited resistance to at least one antimicrobial agent, revealing a significant variability with 42 different antimicrobial resistance profiles. Although no XDR or PDR isolates were detected, 18.18% of the strains were classified as MDR. While this percentage may seem modest, these findings underscore the growing concern about antimicrobial resistance, particularly given that the sampled staphylococci originated from wild animals inhabiting a natural park that may come into contact with antimicrobials or resistant bacteria in contaminated environments. Data on MDR staphylococci in deer are limited; however, previous studies have reported a lower prevalence of MDR isolates [22,25,47]. One possible explanation for the observed resistance is the proximity of the park to high-density human areas and farms that raise horses, bovines, small ruminants, and avian species. The park is also a popular destination for recreational activities, featuring amenities such as a hotel, restaurant, a hippodrome, and a reproductive center for horses. The presence of extensive breeding of cattle and horses within the park further increases the likelihood of interaction between domestic and wild animals, potentially facilitating the transmission of resistant strains. In this study, most MDR strains were identified as *S. sciuri*, a coagulase-negative *Staphylococcus* typically regarded as opportunistic. Nevertheless, research has highlighted the significance and potential impact of *S. sciuri* on both humans and domestic animals [58]. Therefore, monitoring the carriage of staphylococci other than *S. aureus* in wildlife could be essential for identifying potential reservoirs of antimicrobial resistance.

Finally, we examined the presence of specific virulence genes, particularly those responsible for enterotoxins and toxic shock syndrome toxins. These virulence factors were chosen due to their significant implications for human health. In fact, the recent report from EFSA and ECDC highlighted that in Europe in 2022, toxins from *S. aureus* were the second most frequently reported cause of foodborne outbreaks attributed to bacterial toxins and the leading cause of hospitalizations and fatalities [16]. We chose not to investigate additional virulence factors since the staphylococci under consideration were derived from healthy animals. Our findings suggest that fallow deer do not serve as carriers of potentially toxigenic staphylococci, as all the strains tested negative for the targeted virulence genes. This outcome aligns with previous studies on wildlife. For instance, a recent review that evaluated the role of wildlife as a reservoir for *S. aureus* referenced approximately 20 studies on various wild animals across Europe, none of which reported the presence of enterotoxin genes in isolated strains [59]. Likewise, a recent investigation focusing on *S. aureus* strains from hedgehogs in Hungary did not find any staphylococcal enterotoxin (SE) genes [23]. In contrast, two studies conducted in Poland reported a 14.9% and 70% positivity rate for SE genes in *S. aureus* strains isolated from wild boar and red fox, respectively [60,61]. Additionally, research from Spain detected SE genes in *S. aureus* in 21.31% of strains isolated from various wild animals, including wild ruminants [22].

## 5. Conclusions

The findings from this study underscore the significant role that wildlife, particularly deer, play as a reservoir of antimicrobial-resistant and multidrug-resistant staphylococci. Our research confirms that *S. aureus* is a prevalent inhabitant of the nasal cavity of deer while also highlighting the diverse array of staphylococcal species present in the nostrils of fallow deer. Nearly all identified bacterial species are known as potential pathogens for humans, pets, and livestock. Although the percentages of antimicrobial-resistant and MDR strains found are relatively low, our results indicate the circulation of these resistant staphylococci in wildlife that have not been exposed to antimicrobial treatments. This suggests environmental dissemination and raises concerns about the broader implications of antimicrobial resistance. Notably, we detected several strains of *S. sciuri* exhibiting high resistance levels, pointing to the possibility of fallow deer acting as reservoirs and spreaders of this pathogen. These wild ruminants may serve as a transmission source for other animals in their habitat, as well as for humans, particularly hunters, veterinarians, and park personnel who interact with wildlife. Therefore, ongoing monitoring of wildlife across various geographic regions is vital for managing the spread of antimicrobial resistance and for early identification of potential threats.

## Figures and Tables

**Table 1:** PCR primers employed for the detection of virulence and antimicrobial resistance genes.

Gene	Primer	Oligonucleotide Sequence (5′-3′)	Annealing Temperature	Amplicon Size (bp)	Reference
*sea*	SEA-3	CCTTTGGAAACGGTTAAAACG	55 °C	127	[40]
	SEA-4	TCTGAACCTTCCCATCAAAAAC			
*seb*	SEB-1	TCGCATCAAACTGACAAACG		477	
	SEB-4	GCAGGTACTCTATAAGTGCCTGC			
*sec*	SEC-3	CTCAAGAACTAGACATAAAAGCTAGG		271	
	SEC-4	TCAAAATCGGATTAACATTATCC			
*sed*	SED-3	CTAGTTTGGTAATATCTCCTTTAAACG		319	
	SED-4	TTAATGCTATATCTTATAGGGTAAACATC			
*see*	SEE-3	CAGTACCTATAGATAAAGTTAAAACAAGC		178	
	SEE-2	TAACTTACCGTGGACCCTTC			
*tst1*	TST-3	AAGCCCTTTGTTGCTTGCG		447	
	TST-6	ATCGAACTTTGGCCCATACTTT			
*mecA*	MecA147-F	GTGAAGATATACCAAGTGATT	50 °C	147	[44]
	MecA147-R	ATGCGCTATAGATTGAAAGGAT			
*mecC*	mecLGA251 f	GCTCCTAATGCTAATGCA	50 °C	304	[45]
	mecLGA251 r	TAAGCAATAATGACTACC			
*blaZ*	blaZ-F	CAGTTCACATGCCAAAGAG	50 °C	772	[41]
	blaZ-R	TACACTCTTGGCGGTTTC			
*ermA*	ermA-F	TCTAAAAAGCATGTAAAAGAA	52 °C	645	[42]
	ermA-R	CTTCGATAGTTTATTAATATTAG			
*ermB*	ermB-F	GAAAAGTACTCAACCAAATA		639	
	ermB-R	AGTAACGGTACTTAAATTGTTTA			
*ermC*	ermC-F	TCAAAACATAATATAGATAAA		642	
	ermC-R	GCTAATATTGTTTAAATCGTCAAT			
*msr(A/B)*	msr(A/B)-F	GCAAATGGTGTAGGTAAGACAACT	55 °C	399	[42]
	msr(A/B)-R	ATCATGTGATGTAAACAAAAT			
*mph(C)*	mphC-F1	ATGACTCGACATAATGAAAT	45 °C	900	[41]
	mphC-R1	CTACTCTTTCATACCTAACTC			
*aac(6′)- Ie-*	aac(6′)- Ie-	CCAAGAGCAATAAGGGCATA	60 °C	220	[43]
	aph(2″)- Ia_F				
*aph(2″)- Ia*	aac(6′)- Ie-	CACTATCATAACCACTACCG			
	aph(2″)- Ia_R				

**Table 2:** *Staphylococcus* species isolated from the analyzed fallow deer in relation to the animal age.

Staphylococcus Species	Animal Age						Total of Isolates	
	Young (<1 Year)		Juvenile (>1 and <2 Years)		Adult (>2 Years)			
	Number	%	Number	%	Number	%	Number	%
*S. aureus*	27	40.30	5	33.33	34	36.17	66	37.50
*S. hyicus*	20	29.85	1	6.67	13	13.83	34	19.32
*S. sciuri*	5	7.46	8	53.33	19	20.21	32	18.18
*S. chromogenes*	6	8.96	0	0.00	21	22.34	27	15.34
*S. xylosus*	4	5.97	1	6.67	6	6.38	11	6.25
*S. warneri*	4	5.97	0	0.00	1	1.06	5	2.84
*S. devriesei*	1	1.49	0	0.00	0	0.00	1	0.57
Total	67		15		94		176	

**Table 3:** Results of the antimicrobial susceptibility test.

	Susceptible		Intermediate		Resistant	
	N°	%	N°	%	N°	%
Penicillin	124	70.45	0	0.00	52	29.55
Amoxicillin–clavulanate	175	99.43	0	0.00	1	0.57
Cefoxitin	172	97.73	0	0.00	4	2.27
Ceftiofur	131	74.43	37	21.02	8	4.55
Chloramphenicol	147	83.52	21	11.93	8	4.55
Tetracycline	153	86.93	18	10.23	5	2.84
Enrofloxacin	107	60.80	50	28.41	19	10.80
Ciprofloxacin	127	72.16	35	19.89	14	7.95
Gentamicin	154	87.50	10	5.68	12	6.82
Amikacin	140	79.55	0	0.00	36	20.45
Trimethoprim–sulfamethoxazole	154	87.50	8	4.55	14	7.95
Erythromycin	58	32.95	109	61.93	9	5.11
Rifampicin	119	67.61	17	9.66	40	22.73

**Table 4:** Antimicrobial resistance profiles in relation to the staphylococcal species.

	Number of Isolates							
Resistance Profile	*S. aureus*	*S. chromogenes*	*S. hyicus*	*S. xylosus*	*S. sciuri*	*S. warneri*	*S. devriesei*	TOT
No resistance	37	17	17	5	10	4	1	91
AK	3	1	1					5
P	1	4 #^(2)^	10	3	3			21
CIP	1							1
EFT			1		1			2
RD	5	1			1			7
SXT	1							1
AK-SXT	1							1
P-EFT			1					1
P-RD	1		1	1	3			6
P-SXT				1 #				1
CIP-AK	1							1
ENR-AK	1					1		2
ENR-CIP		1						1
ENR-RD	1							1
P-AMC-FOX			1					1
CN-AK-SXT	1							1
P-AK-RD *	1				4			5
P-E-RD *					1			1
P-ENR-E *	1							1
TE-AK-RD *	1							1
P-AK-SXT-RD *	1				1			2
P-CN-AK-RD *					1			1
P-EFT-AK-RD *					1			1
P-ENR-AK-RD *					1			1
P-ENR-CIP-RD *	1							1
C-CN-AK-E *		1						1
CIP-CN-AK-RD *	1							1
EFT-C-CN-AK *			1					1
ENR-CIP-AK-RD *	1							1
P-EFT-ENR-AK-RD *					1			1
P-ENR-CIP-SXT-RD *			1					1
P-ENR-CN-AK-RD *					1			1
P-FOX-ENR-CIP-RD *					1			1
P-C-TE-CN-AK-SXT *	1							1
P-CIP-CN-AK-SXT-RD *	1							1
P-EFT-C-CN-AK-RD *					1			1
P-ENR-CIP-AK-SXT-RD *	1							1
FOX-EFT-ENR-AK-E-RD *					1			1
FOX-TE-ENR-SXT-E-RD *	1							1
C-ENR-CIP-CN-AK-SXT-E *		2						2
P-C-TE-ENR-CIP-AK-E-RD *				1				1
C-TE-ENR-CIP-CN-AK-SXT-E-RD *	1							1
Total	66	27	34	11	32	5	1	176

Legend: P = penicillin, AMC = amoxicillin–clavulanate, FOX = cefoxitin, EFT = ceftiofur, C = chloramphenicol, TE = tetracycline, ENR = enrofloxacin, CIP = ciprofloxacin, CN = gentamicin, AK = amikacin, SXT = trimethoprim–sulfamethoxazole, E = erythromycin and RD = rifampicin; * = MDR; # = *blaZ* positive isolates.

## Data Availability

The original contributions presented in the study are included in the article, further inquiries can be directed to the corresponding author.
